# Evaluation of Prompted Annotation of Activity Data Recorded from a Smart Phone

**DOI:** 10.3390/s140915861

**Published:** 2014-08-27

**Authors:** Ian Cleland, Manhyung Han, Chris Nugent, Hosung Lee, Sally McClean, Shuai Zhang, Sungyoung Lee

**Affiliations:** 1 School of Computing and Mathematics, Computer Science Research Institute, University of Ulster, Newtownabbey, Co. Antrim, BT38 0QB, Northern Ireland, UK; E-Mails: cd.nugent@ulster.ac.uk (C.N.); s.zhang@ulster.ac.uk (S.Z.); 2 Ubiquitous Computing Laboratory, Kyung Hee University, Seocheon-dong, Giheung-gu 446-701, Korea; E-Mails: smiley@oslab.khu.ac.kr (M.H.); hslee@oslab.khu.ac.kr (H.L.); sylee@oslab.khu.ac.kr (S.L.); 3 School of Computing and Information Engineering, University of Ulster, Coleraine, Co. Londonderry, BT52 1SA, UK; E-Mail: si.mcclean@ulster.ac.uk

**Keywords:** activity recognition, ground truth acquisition, experience sampling, accelerometry, big data, mobile sensing, participatory sensing, opportunistic sensing

## Abstract

In this paper we discuss the design and evaluation of a mobile based tool to collect activity data on a large scale. The current approach, based on an existing activity recognition module, recognizes class transitions from a set of specific activities (for example walking and running) to the standing still activity. Once this transition is detected the system prompts the user to provide a label for their previous activity. This label, along with the raw sensor data, is then stored locally prior to being uploaded to cloud storage. The system was evaluated by ten users. Three evaluation protocols were used, including a structured, semi-structured and free living protocol. Results indicate that the mobile application could be used to allow the user to provide accurate ground truth labels for their activity data. Similarities of up to 100% where observed when comparing the user prompted labels and those from an observer during structured lab based experiments. Further work will examine data segmentation and personalization issues in order to refine the system.

## 1. Introduction

Smartphone ownership has increased dramatically since being first introduced nearly a decade ago. Modern smartphones are now equipped with various inbuilt sensor technologies, including GPS, accelerometry, light sensors and gyroscopes, large memory storage, fast processing and lower power communications, which allow them to meet the requirements of the range of data to be collected [1]. Furthermore, many people already own smart phones, are accustomed to carrying them and always keep them charged. For these reasons smartphones are viewed as being well suited for use as a mobile sensing platform. Indeed, participatory and opportunistic sensing, leveraging the user's own mobile device, to collect social, physiological or environmental data, is gaining popularity [1,2]. One application area which has been extensively studied over recent years is that of activity recognition (AR). AR is concerned with the automatic recognition of a user's activity using computational methods. These activities can include low level activities such as walking or sitting, in addition to higher level activities such as grooming or cooking. AR has many potential applications including, activity promotion, self-management of chronic conditions, self-quantification, life logging and supporting context aware services. From a data driven perspective, the development of automatic AR techniques is achieved through the application of machine learning techniques to data gleaned from low level sensors, such as those found on a smart phone [3]. The training of these algorithms relies largely on the acquisition, preprocessing, segmentation and annotation of the raw sensor data into distinct activity related classes. For this reason the data must therefore be labeled correctly prior to being used as a training set within the data driven machine learning paradigm [4]. These algorithms are normally trained and tested on data from a small number of participants under closely supervised conditions, which may not reflect those of free living conditions [5]. Training using sensor data collected on a large scale and under free living conditions has the potential to improve the generalizability of any AR models. Indeed, a large scale data set is recognized as being a key step in improving and increasing the widespread adoption of AR based applications [6,7]. Such large scale data sets should also include data from a variety of sensors, recorded during a wide range of activities and contexts from a large number of users, over an extended period of time (months or even years). Most importantly the data should also include accurate ground truth labels that represent user activities [8].

This paper details an evaluation of a smart phone based data labeling application which prompts the user to provide accurate ground truth labels for sensor data for the purposes of creating a data set to be used to generate data driven AR models. The application aims to overcome the challenges associated with collecting annotated activity data on a large scale in free living conditions. Prompting the user, based upon their activity transitions as detected by an underlying AR module, provides a novel way of capturing accurate data labels on a large scale. In order to provide further context for this work, a review of related works is provided in Section 2. Following on from this the system architecture of the prompting application is described followed by the protocol for the evaluation. The paper concludes with a discussion of the results from the evaluation and scope for further work.

## 2. Background

**Figure 1.**
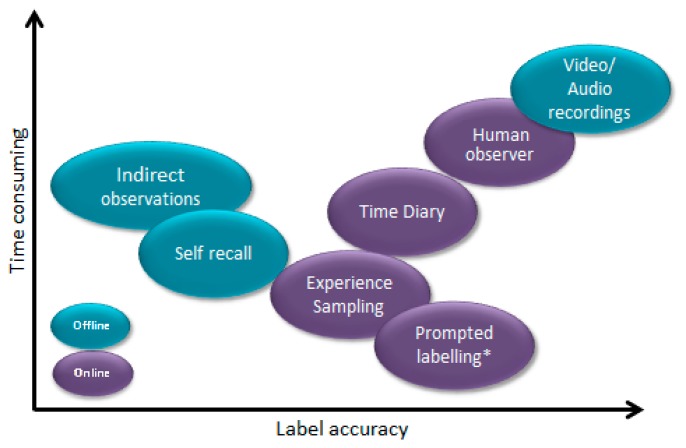
Common methods of ground truth acquisition, highlighting the tradeoff between time required and label accuracy. Figure has been redrawn from [13]. Prompted labeling denotes the method proposed within this paper.

A large amount of research has focused on the ability to accurately recognize a range of activities. These studies have utilized data from wearable sensors [9,10] and those found within smartphones [11,12] and have addressed a number of application areas [4]. Very few studies have, however, provided a detailed description of how the ground truth of data sets, for the purposes of a data driven approach, have been acquired. Methods of obtaining ground truth can be carried out both online or offline [13]. Figure 1 highlights the common methods of both online and offline ground truth acquisition. To date the majority of AR studies have used data collected under structured or semi-structured conditions, from a small number of participants (1–20 subjects). In these instances, participants perform a set of preplanned tasks which are completed within a controlled environment [14–17]. The ground truth is often recorded by a human observer and annotated offline. This is deemed to be essential as it allows researchers to capture the ground truth, in order to label the data, in an effort to create highly accurate data sets. Data collected in this manner may not, however, be truly representative of completing activities in a free living environment, given that it lacks the natural variations that would be apparent when collected in a free living environment. Boa and Intille asked participants to complete a list of planned activities and to note the time at which they started and completed each activity [17]. This process of continuously recording the time at which an activity commenced and was completed is suitable for short term laboratory based studies, however, would not be feasible over longer periods of time in free living conditions where it can become intrusive and disruptive to the user's daily activities. Furthermore, processing and labeling data in this manner can be a laborious and time consuming task for researchers, particularly if collecting data from a large number of participants. When dealing with large numbers of participants and/or over long periods of time, it is also not practical or feasible to employ a human observer to follow multiple participants.

In order to allow the collection of data in a free-living environment, researchers have utilized video cameras [18]. The subsequent video recording is then reviewed offline to identify what activity was being performed at a particular point in time. Similar techniques have been used within smart environments to label the onset/completion of object interactions [19]. Again, however, this process is labor intensive and time consuming, particularly for a large number of participants as each recording has to be reviewed and annotated. Some researchers have attempted to deal with these labor intensive tasks by using groups of labelers sourced from the cloud. Lasecki *et al.* [20] used activity labels, generated by groups of crowd sourced labelers to annotate activities from video data.

All of the aforementioned methods of obtaining the ground truth labels are labor intensive and time consuming. Furthermore, some approaches, in particular those associated with video annotation, may have implications for data privacy. Additionally, the need to install or issue video cameras for recording the activities reduces the scalability of such an approach.

For larger scale studies, users may be asked to annotate their own data using an interface on a mobile device. This requires the user to start and stop the data capture process manually [21]. Whilst using the application the user is then asked to label the activity they have just or are about to complete. Although this method is relatively accurate for segmenting the activity it requires the user to explicitly start and stop the recording. Other studies have used time constraints in order to periodically prompt the user to provide information on what activity they are doing. Tapia *et al.* [22] used a technique based on the experience sampling method to trigger self-reported diary entries every 15 min. Multiple choice questions were answered by the user to determine which of 35 activities they had just completed. Due to the intermittent nature of the labels it was found to be difficult to detect short time related activities. Furthermore, as with other methods, the process of continually labeling data can become laborious for users, particularly when carried out over an extended period of time. This can result in the user providing incorrect labels for the data or simply not engaging with the system at all. In addition, in order for the user to input a label, some interaction with the mobile device is required. This may interrupt the user during the activity, which in turn may impact on the activity that the person is undertaking, thus impacting overall on the data recorded. In an attempt to address the issue of interaction voice recognition has been used for the purposes of annotation [23]. The mobile device listens for key words such as “Start activity” to start and stop the recording. Voice recognition is then used to label the activity, with the user again saying keywords, such as “standing” or “walking”. Nevertheless, having the smart phone continuously listening for keywords can consume battery power and may hamper the usability of the application. Additionally, inaccuracies of voice recognition can lead to mislabeling of data.

Systems designed to collect labels for activity data on a large scale rely primarily on time based experience sampling or video annotation data. These systems have a number of limitations in relation to the labour intensity and intrusive nature of the systems. The current approach, discussed in this paper, uses prompted labeling, driven by an underlying mobile based AR module, in an effort to improve the process of collecting and annotating data sets. Users can annotate their everyday activities through the use of a personalized mobile application. When the user is detected as standing still, a prompt is provided to enable the user to label the activity they were previously completing. In this way the sensor data for the respective activity is segmented and saved automatically and a ground truth label is supplied by the user after the activity has finished thus maintaining the integrity of the data. Previously algorithms for activity recognition have relied on data collected under strict conditions, however, this data may not be representative of activity data collected in a real world situation on a large scale. As the app is to be used in a free living environment, where there is no reference of a human observer or camera and where users do not follow scripted activities, the most appropriate way to find out what the user is doing is to ask them. A number of studies take the approach of requesting that the user provide ground truth information with which to annotate their data [17,20–23]. The current method, however, is the first to use change in activity to prompt a user for this information, most previous solutions use only temporal information. Methods of collecting data on a larger scale within free living conditions have largely focused on time based or random (experience sampling) prompts. These methods may not, however, produce accurate labelling as described above. The contribution presented within this work is the design and evaluation of a context aware method to collect ground truth labels for activity data within a free living environment based on change in activity. The ability to reliably collect and efficiently annotate training data has been highlighted as a critical challenge in enabling activity recognition on a large scale in free-living conditions [24]. The proposed method extends previous works, by providing a more intelligent, context aware, method of prompting the user, rather than simply temporal based. The authors believe this may make it possible to provide a higher accuracy of labeling whilst reducing the potential of interrupting the user during an activity. Collecting such data on a large scale will allow the accuracy of current activity recognition methods to be improved whilst expanding upon the types of activities which can be recognized. The appropriate evaluation of the proposed solution is an important stage within the development as it provides a solid foundation in which to produce better quality, fully annotated datasets which can then be used to create more accurate activity recognition models.

## 3. System Architecture

**Figure 2.**
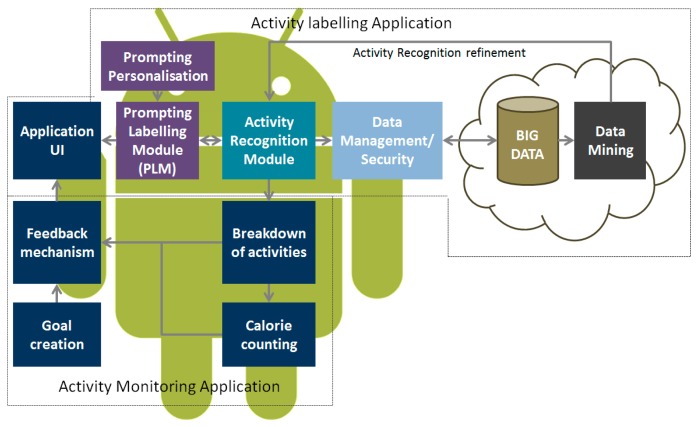
Overview of personalized mobile application for prompt labeling. The prompted labeling module sits on top of an existing AR module and periodically prompts users to label their activity. The architecture includes mobile services to support the secure transmission and processing of data in addition to the collection of other sensory data available from the mobile platform.

This Section provides details of the system architecture. The mobile application is based upon the principle of prompts to label a user's context and activity data. At periodic times throughout the day, the application will prompt the user to ask them to indicate which activities they have just completed. These prompts are based upon the AR module which prompts the user to label their activity when the activity standing still is detected. In addition to user reported data, additional information gleaned from the mobile device, such as automated activity classifications, GPS latitude and longitude, accelerometry data and Bluetooth interactions are also recorded. This additional data aids in further contextualizing the annotated data sets with the intention of improving the validity of labeling. An overview of the system architecture is presented in Figure 2. The application was implemented on the Android operating system and was tested on a range of handsets including Nexus 5 and Samsung Galaxy S3 and S4.

### 3.1. Activity Recognition Module

The AR model within this work, developed by Han *et al.* [24], utilizes multimodal sensor data from accelerometery, audio, GPS and Wi-Fi to classify a range of everyday activities such as walking, jogging and using transport. The AR is carried out independently of the position or orientation of the smart phone. This has the effect of increasing the practicality and usability of the system, as the phone can be carried in a variety of locations. Data from the accelerometer is used to detect transitions between ambulatory activities to activities which involve the use of transport, *i.e.*, riding a bus. Accelerometer data, sampled at 50 Hz, is computed into time and frequency domain features which are subsequently used as inputs to a Gaussian Mixture Classifier. Audio data is used in the classification if there is a need to classify between transportation activities (taking a bus or subway). By only using the audio when necessary allows the power consumption on the smart phone to be minimized. GPS and Wi-Fi signals are then used to validate the classification between activities. Speed information, derived from GPS is used to determine whether a user is walking, running or standing still. The Wi-Fi signal is used to differentiate between bus and subway activities, as very few public or private wireless networks are available within the subway system. Full details of the AR module, including details of evaluation and accuracy can be found in [25].

### 3.2. Prompted Labelling Module

**Figure 3.**
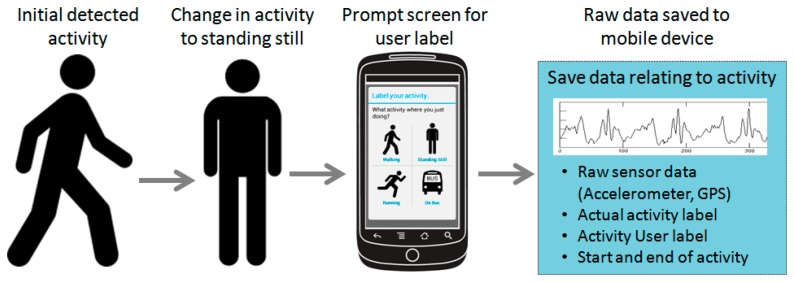
An example of the user interaction with the prompt labeling screen. The AR module detects a change in class from the original activity to standing still. The prompt is then issued for the user to label their previous activity. Raw sensor data is then saved to the mobile device before being uploaded to the cloud for further processing and storage.

The prompted labeling module (PLM) prompts the user to provide a label for the activity they have just completed. Based on the output from the AR module, the PLM polls for class transitions from any of the activities (for example walking or running) to the standing still activity. Once a transition has been detected the PLM prompts the user, through the provision of an audio and vibration alert on the smart phone, to provide a label for the last activity that was undertaken. The raw data from the accelerometry sensor is then stored on the mobile device before being transmitted to the cloud for processing and storage. By prompting the user to label the activity we can verify that the activity has been correctly identified by the AR module. In this way the validity and the trustworthiness of the AR module can be tested in addition to providing a fully annotated data set. Figure 3 presents an example of the interaction with the prompt labeling screen on the mobile device in addition to a screen shot of the mobile application's interface.

**Figure 4.**
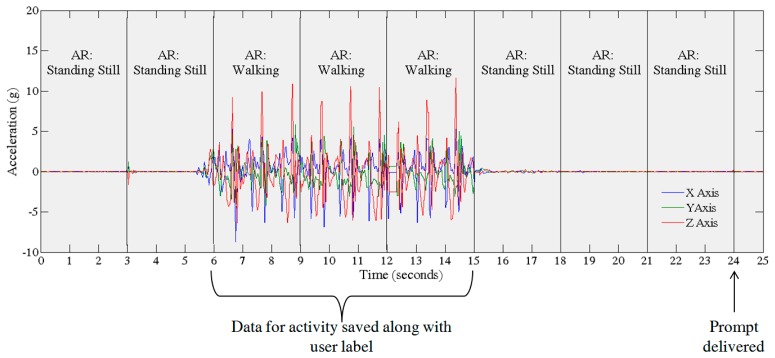
Illustrates how activities are detected from the raw accelerometer signal by the AR module. Activities are detected every 3 s; three consecutive detections are used to label the activity. The prompt is initiated when the AR module detects a change in class from one activity to standing still.

The AR module detects an activity based on three seconds (150 samples) of data. Three consecutive detections (9 s) are then used to label the activity. This is carried out in order to limit the number of detection errors. Once the AR module detects a change from the current activity to the Standing Still activity for 9 s the previous activity data from the sensors is saved to memory. This process, from the perspective of raw accelerometry data is depicted in Figure 4. Currently, the prompt is initiated every time the AR module detects a transition from an activity to standing still.

Currently data, sampled at 50 Hz, recorded by the system is stored directly to local memory, in the form of a text file. Data recorded includes date and time stamp, raw accelerometer values (*X*, *Y* and *Z* axis), GPS latitude and longitude in addition to the class label from the AR module and the label recorded by the user. For the purposes of evaluation the details of the time taken for the user to answer the prompt were also stored. Following 20 s of no user interaction the prompt message is removed and the prompt is recorded as missed. 20 s was chosen as an appropriate length of time for a user to answer the prompt without impacting on subsequent notifications. This timeframe was tested empirically, with two people 10 times, during the design of the app itself. Furthermore, studies have shown that the majority of activities occur in short bouts (<30 s) [26]. Other works utilizing experience sampling techniques have shown response times of 20–30 s, with users interacting with their smartphone for less than 30 s 50% of the time [27,28].

## 4. Experimental Methods

In order to evaluate the accuracy of the prompting method to gather ground truth labels, we needed to compare the labels provided from the user with the Gold standard/reference of labels provided by the human observer. Human observers are commonly used in many activity recognition studies as the source of ground truth [13]. The evaluation of the proposed solution consisted of three separate experimental protocols. These were chosen in order to collect data relating to app usage and the accuracy of labelling during a range of contexts. The first (Task 1) was a structured laboratory based experiment which sought to assess the precision of the user specified label when performing a timed set of activities. The second (Task 2) uses semi structured tasks, in which the participant is observed whilst carrying out tasks and using the device. The third (Task 3) is based on free living where the user uses the system whilst going about their daily life; during this stage there is no human observer.

### 4.1. Evaluation Protocol

Ten participants were recruited from Kyung Hee University to participate in the study. All participants were free from motor impairments which may have affected their ability to carry out the prescribed tasks. Participants were also owners of an Android smart phone and were familiar with its use. The evaluation protocol was split into three separate experiments in order to assess the accuracy of labeling and usability of the system. Details of each of the experiments are provided in the following sub-sections.

#### 4.1.1. Task 1: Timed Lab Based Experiment

The timed lab based experiment required the participant to carry out a number of activities for a set period of time. During this time the participant was observed by a researcher whilst completing the protocol. Timings for each of the activities were kept by the observer. Prior to commencing the experiment the prompted labelling application was installed on the user's smart phone. The application was subsequently started and the participant was asked to place the device in the location where they normally kept it (Pocket, Bag, *etc.*). A human observer recorded the location of the mobile phone. The participant was then asked to complete the activities presented in Table 1. During this time, when the participant transitioned from any of the activities back to standing still, the mobile application prompted the participant to label their previous activity. When this occurred the participant was instructed to take the mobile phone from the location it was being carried and answer the prompt. Following this the participant replaced the phone in the same location.

#### 4.1.2. Task 2: Semi-Structured Evaluation Protocol

The second experiment was based upon a semi-structured protocol which aimed to simulate a free living environment. In this protocol the participant was asked to carry out tasks while followed by an observing researcher. In this case, however, the participant was not instructed how or for how long the activity should be carried out. Instead the participant was asked to complete a set of tasks as detailed in Table 2. These tasks alternated between a range of activities including; Walking, Standing still, Jogging and taking a Bus. Again the user was instructed to remove the mobile device from where it was being kept when they felt the application prompted them to label their activity. The human observer noted the time at which an activity started and was completed and also noted the times at which the application prompted the user to label their activity. This information was used as the gold standard.

#### 4.1.3. Task 3: Free Living Experimental Protocol

The final protocol focused on a free living experiment. This allowed the participant to use the app whilst going about their everyday activities. The aim of this was to collect information on how the system would be used in a free living environment. The user was asked to carry their mobile device, with the mobile application installed, for a full working day (or for as long as possible). The participant was instructed to carry and interact with the device as they would normally. The mobile application would then prompt the user to label their activity throughout the day. Accelerometer data related to the participant's activity in addition to labels provided both by the participant and generated by the AR module were recorded. Other metrics such as the time taken to answer a prompt and when prompts where missed was also recorded. On completing the experiment the participants were then asked to complete a post task questionnaire on their feelings towards how the app functioned and general questions in relation to usability. Usability questions were based upon a customised version of the IBM Computer Usability Satisfaction Questionnaire (CUSQ) along with questions specific to the prompting of the application *i.e.*, did the system prompt too often, did you find it intuitive to label your activity data [29]. Answers to questions were recorded on a seven point Likert scale, with 7 being strongly agree and 1 being strongly disagree. The CUSQ is separated into 4 sections; Overall satisfaction score (OVERALL: all 18 questions), system usefulness (SYSUSE: questions 1–8), information quality (INFOQUAL: questions 9–15) and interface quality (INTERQUAL: questions 16–18). In addition to the 18 questions of the CUSQ participants were also asked about where the phone was carried and frequency of prompting.

## 5. Results

Of the ten users who participated in the study nine completed all three tasks of the evaluation protocol. One participant failed to complete the free living segment of the evaluation. This was due to a technical fault with the application causing it to force close and failing to record the data from the evaluation. The following Sections present details of the frequency with which prompts were delivered and answered, the accuracy of the labels provided by the AR module and from the user via the prompting application and how the participants felt about the usability of the app.

### 5.1. Prompting Frequency

On average it took the participants 4.2 s to answer the prompt during the first evaluation protocol, 6.11 s during the semi-structured protocol and 7.77 s during the free living protocol. This was to be expected given that during free living participants would not necessarily be expecting the mobile device to prompt them to label their activity. Participants received 3 to 4 prompts during the first protocol, 4 to 5 prompts during the semi-structured protocol and between 6 and 56 prompts during the free living protocol. Results for each participant are presented in Table 3. Again an increase in the number of prompts was to be expected given the increased time spent using the application during Task 3 and therefore the increased number of transitions to standing still was detected by the application. Participants spent on average 22 min completing the semi-structured protocol, compared to 4 h 11 min on average using the app under free living conditions. The participants missed no prompts during Tasks 1 and 2 of the evaluation protocol. This illustrates that it would be feasible to prompt a user to label their activity based on this transition method. Task 3 was the only task where users missed prompts with 34/237 missed prompts in total. These missed prompts were not included when calculating the average time to answer the prompt.

### 5.2. Comparison between Labels

In order to assess the accuracy of labeling, labels provided by the AR module and from the participants in response to the prompt, were compared to the labels provided by the human observer in addition to each other. Labels from a human observer are viewed as the gold standard for labeling activity data. For Task 1 and 2 the prompted label provided by the user and the label from the observer had the highest similarity with the labels being the same on average 100.0% of the time during Task 1 and 97.96% of the time during Task 2. A summary of the comparison of the results is provided in Table 4.

The labels from the AR module were the least accurate source of labels, with the AR label being the same as that from the human observer 86.84% of the time for Task 1 and 85.71% of the time for Task 2. This demonstrates that prompted labeling can be used to accurately label data during structured and semi-structured settings. As there was no human observer for Task 3 the AR prompt was compared only to the prompted label provided from the user. For Task 3 the label provided from the AR module was the same as that provided by the user when prompted 30.54% of the time. This low agreement was mainly due to the AR module confusing the activity of riding a bus with the standing still activity, this occurred in 30 instances.

### 5.3. Usability Questionnaire Results

The CSUQ was completed by the nine participants who completed all three tasks of the evaluation. Participants carried the mobile phone in a range of locations including their trouser pocket (four in the right pocket and one in the left pocket), jacket pocket and bags. Participants found the system simple to use, rating it on average 5 out of 7 for overall usefulness. Average system usefulness was rated 5.3, information quality 4.7 and interface quality 4.6. Table 5 presents a summary of the results from the CSUQ.

Two of the nine participants admitted to missing a prompt, and gave the reasons that they were too busy to answer the prompt or did not hear the alarm tone. The participants felt that the app had prompted them to label their activity too often, with one participant noting that the app had interrupted them whilst carrying out an activity. It is worth noting that when asked about the negative aspects of the application, six out of the nine participants mentioned misclassification of activities; the activity of riding a bus was commonly misclassified as standing still. This highlights that improvements in classification accuracy is crucial to application usability.

## 6. Discussion

Results indicated that using a prompting approach, based on transitions to standing still from any of the other activities, allowed users to provide accurate labels for their own activity data. The labels from the AR module were similar to those provided by the user in response to the prompt during the structured and semi-structured protocols Tasks 1 and 2. Nevertheless, during Task 3, the free living task, the similarity between the AR and the prompted label decreased significantly. This shows that even though the AR module worked accurately during structured lab based experimentation, it was not capable of dealing with a more real life setting. This provides further evidence to support the need for a large scale annotated dataset recorded under free living conditions in order to train, test and subsequently improve existing AR modules. Labeled data collected in a free living environment, using methods such as those described in this paper, will provide opportunities to improve AR models through the process of active learning. Active learning focuses labeling efforts on instances with the most error, asking the participant to provide labels for data which the recognition module is unsure about [30]. This new labeled data can then be fed back into the AR module in order to retrain and to better recognize the instance. Online processing of data in this way is, however, computationally demanding, particularly when being carried out in real time on a mobile device. Nevertheless, it may be possible to process this data offline, transmitting data to the cloud for processing, retraining and updating the model before updating on the mobile device.

**Figure 5.**
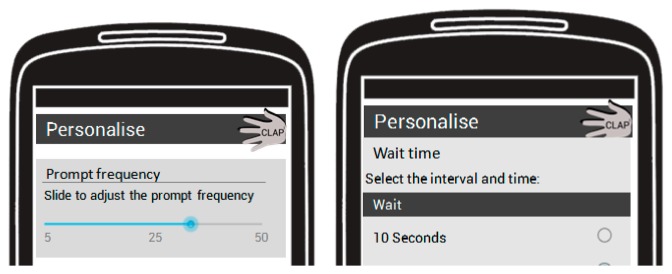
Screen shots of proposed personalization features allowing the user to set the frequency of the prompt and how long the system allows them to respond to a prompt.

Participants found labeling their data using the app both simple and intuitive. It was noted, however, that the app prompted the participants too often. In this evaluation the app prompted the user to label their activity every time a transition to standing still was detected by the AR module. This led to the user experiencing multiple prompts in a relatively short period of time. This could lead to the user becoming irritated by the application and therefore no longer using it. It is therefore critical that some level of personalization of the app is provided allowing the user to control the frequency of the prompts or how many prompts they answer each day. A visual representation of what this personalization may look like is provided in Figure 5. The app may also allow the user to set the time or location based constraints on when they receive prompts, for example not receiving a prompt after 9 p.m. or not receiving a prompt whilst they are at work. This customization would again provide the user with the opportunity to control when and where they are prompted thus lessening the chances of them becoming irritated by the application. The impact of this will be investigated through a larger scale evaluation which will also be important in assessing application robustness.

Furthermore, participants noted that they occasionally missed prompts due to not hearing the notification tone or being too busy to respond to the prompt. Again this can be partially addressed through personalization allowing the user to select the time provided to respond to the prompt and to select/adjust the notification sound.

**Figure 6.**
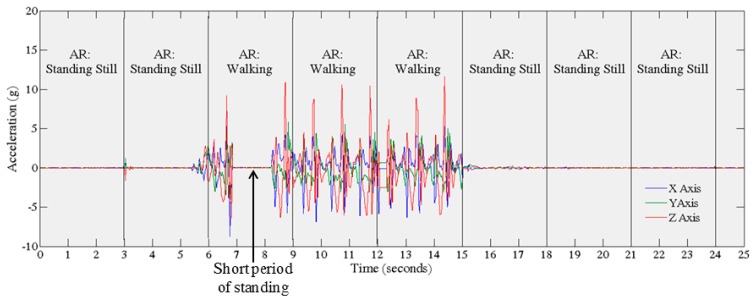
Illustrates the potential issue where data labeled as walking by the AR module and the user may in fact include intermittent data from walking and another activity, in this case standing still.

The current application was developed through an iterative design process. Initially it was surmised that detecting activity change points using the output from an activity recognition module would provide a fine grained cut-off with which to segment and accurately label the activity. Nevertheless, following testing through this evaluation it was found that this was not the case. Although prompting the user to annotate their data allows for the collection of accurate data labels there are issues around the complexity of data segmentation. For example, in the scenario presented in Figure 6, the user begins walking, stops for a short period of time and then continues to walk. The AR module detects the majority of the window as walking and labels it as such. Nevertheless, if this data, which contains multiple activities, is used for training of an AR module, it may cause the classifier to confuse data from the various activities and therefore lead to reduced classification accuracy. It is therefore important to consider this issue and determine if all the data being saved is in fact representative of the related label.

**Figure 7.**
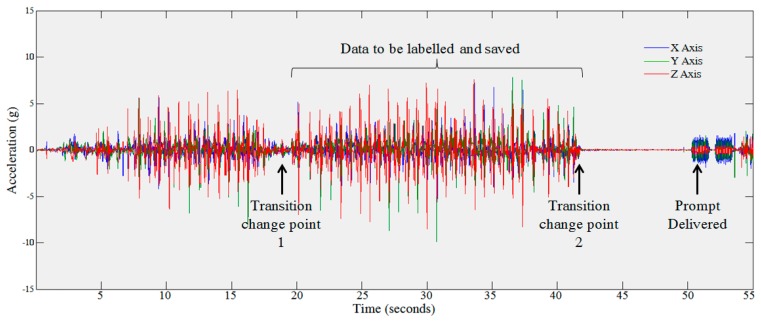
Plot of accelerometry data obtained during experimentation. Two high frequency vibrations can be clearly viewed in the accelerometer trace. This relates to the vibration alert initiated as part of the prompt notification.

A related issue is concerned with detecting the change points associated with transitions from one activity to another. This is illustrated in Figure 7.

Automatically detecting these change points is important as this allows the data to be automatically segmented into distinct classes. This is particularly important when sensing in a real environment, where bouts of activity can occur in very short periods e.g., 10–20 s. The current approach within this app relies upon the AR module to detect the transition point. Therefore, subsequent versions of the app will employ more complex techniques such as Cumulative Sum (CUSUM) and multivariate change detection to improve this process [31]. The online change detection algorithm extends work by [32] to consider multivariate data streams. Initial results on real world data have shown accuracy of 99.81% and precision of 60% when detecting changes in activity [31]. CUSUM [33] is a method of detecting shifts in the mean of a process in order to detect a change point [34]. In this instance CUSUM can be applied to accelerometer data, once a change in pattern is detected, this can be associated with a change in activity. CUSUM has previously been used to detect the differences between activity and inactivity within accelerometer data [35].

A further limitation of the system is the fact that the application only prompts the user to provide a label if the activity is followed by the standing still activity. This means that activities which are not followed by a period of standing still will not be recorded. One way in which to address this may be to prompt the user on a number of “non-obtrusive” activities. These activities would be activities in which the user is least likely to be disturbed by a prompt (such as sitting still or sitting on the bus). Furthermore, it may be possible to delay the prompt to the most convenient time, for example the prompt for the activity could be delivered several minutes after the activity has finished, prompting the user at a convenient time to provide a label for that previous activity. This could be further contextualized by providing time and location information (for example, what activity were you carrying out at 4.00 p.m. at the Supermarket?). Another way in which to reduce the number of prompts per day is to use an active learning approach, as previously described, where the system would only prompt the user to label instances that the AR module is unsure about. This would significantly reduce the number of prompts that the user receives per day whilst simultaneously improving the recognition accuracy. Furthermore, if the application was to prompt continuously throughout the day, this may produce a considerable drain on battery power. By limiting the number of prompts which the user needs to interact with the battery usage of the app can be minimized. Further methods of reducing the computational complexity and improving the power consumption should be investigated within future work, to ensure the system viable on a large scale. Using low power sensors, such as accelerometers is seen as one way to further reduce power consumption. Accelerometers typically have power consumption of 1 mW [36]. Energy consumption of AR can also be reduced by using efficient sampling rates, reducing the sampling rate or turning off the sensors when not required. This is particularly useful for power hungry devices such as audio and GPS sensors [10,37]. Furthermore, reducing the amount of data which is sent to the server, by processing the data on the device using lightweight AR algorithms will also aid in reducing the energy consumption of the AR application. Nevertheless, with many smartphone manufactures now specifying dedicated processors for the purpose of monitoring sensor data and human activity more complex AR techniques are now becoming possible.

**Figure 8.**
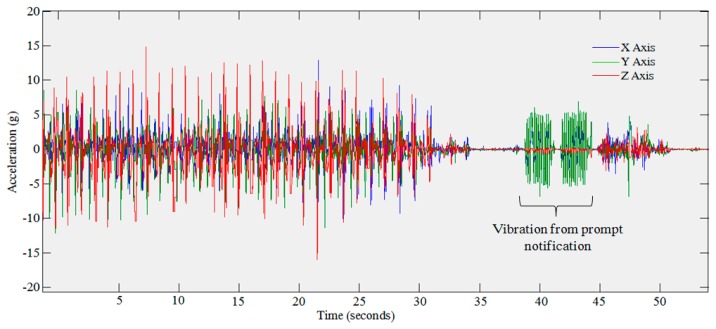
Plot of accelerometry data obtained during experimentation. Two high frequency vibrations can be found in the accelerometer trace. This relates to the vibration alert initiated as part of the prompt notification.

It was noticed whilst analyzing the data from the evaluation that the vibration alert, initiated as part of the prompt, is visible within the accelerometer data. This is clearly visible in Figure 8. This may be of use in later evaluations in order to assess prompt compliance. We can find in Figure 8 that the device was being carried out just before the prompt was delivered and was moved again just afterwards showing that the person was carrying the device when the prompt was delivered. This may provide an insight into the reasons why prompts were missed due to not being carried or if the person was too busy undertaking another task.

Prompting the user to engage with the system whilst stationary may be considered as a reasonable solution to limit the possibility of interrupting the user during an activity. Nevertheless, it still involves the user taking the smartphone from their pocket/bag and providing some interaction. One potential method of reducing this interaction may be to pair the device with a wearable display, such as a smart watch. This would reduce the need for the user to take out their smartphone and may provide a more convenient method of both notifying and capturing labels from the user. With the increased availability and reducing costs of wearable displays this is becoming an increasingly viable solution. Nevertheless, it does require additional hardware which, to date, is not as widely available with consumers as smartphones.

## 7. Conclusions

This paper describes the design and evaluation of a mobile based application which prompts the user to annotate their own activity data based upon the output of an existing AR module. Results indicated that prompting the user provided accurate activity labels which were of similar quality to those obtained from a human observer. There are, however, issues around the automatic segmentation of data, which allow the start and end of the activity to be detected, allowing the data to be clustered into distinct classes. Within a free living environment this can be challenging due to the variable nature of human activity patterns. More complex computational techniques, such as cumulative sum and active learning could be employed in order to address these challenges by providing a more accurate detection of change/transition points within the data. Further issues around the frequency of prompting were also raised, however, it is envisioned that these issues can be addressed through personalization of the application, such as allowing the user to limit the total number or frequency of prompts throughout the day. One limitation of this work is the small number of users with whom the app has been evaluated. Until the app has been fully validated on a larger cohort we cannot be certain as to how effective this method of data labelling will be, particularly in the long term. This, work is however, an important step in assessing the accuracy of the labelling method against the current gold standard of a human observer, which would not be feasible in a larger scale evaluation due to the lack of human observer in this situation. Future work will focus on the creation of cloud services and developing methods for automatic data segmentation prior to testing the application on a larger cohort.

## Figures and Tables

**Table 1.** Activities to be completed during the timed laboratory based experiment protocol (Task 1).

**Task**
Stand still for 30 s
Walk for 30 s
Stand still for 30 s
Jog for 30 s
Stand still for 30 s
Walk for 30 s
Stand still for 30 s
Jog for 30 s
Stand still for 30 s

**Table 2.** Activities completed during the Semi-structured experimental protocol (Task 2).

**Activity Associated with Task**	**Task Description**
Walking	Walk from the office to the Bus stop.
Standing still	Stand and wait for the bus.
Riding Bus	Board the bus and take it to the front entrance of the University.
Standing still	Exit the bus. Stand and read the time table.
Jogging	Jog to the previous bus stop.
Standing still	Stand and wait for the bus.
Riding bus	Ride the bus back to the office.
Standing still	Stop and read something for a while.
Walking	Walk from the bus stop to the office.
Standing still	Stop and read something for a while at the entrance of the office.

**Table 3.** Number of prompts delivered to each participant during each of the three tasks of the evaluation.

**Participant no.**	**Task 1**		**Task 2**		**Task 3**	
		
	**Delivered Missed**		**Delivered Missed**		**Delivered Missed**	
1	4	0	6	0	6	0
2	4	0	6	0	NA	NA
3	3	0	6	0	23	0
4	3	0	5	0	21	0
5	4	0	3	0	6	4
6	4	0	6	0	55	18
7	4	0	5	0	7	1
8	4	0	6	0	6	2
9	4	0	5	0	45	9
10	4	0	6	0	56	0
**Total**	**38**	**0**	**49**	**0**	**203**	**34**

**Table 4.** The number of labels, which were the same as provided by the AR module, prompted from the user and the human observer.

**Task**		**Label from AR *vs.* Prompt Label**	**Label from AR *vs.* Observer Label**	**Prompt Label *vs.* Observer Label**
**Task 1**	**No. Same**	33	33	38
	**% Same**	86.84%	86.84%	100.00%

**Task 2**	**No. Same**	43	44	52
	**% Same**	83.67%	85.71%	97.96%

**Task 3**	**No. Same**	218	-	-
	**% Same**	30.54%	-	-

**Table 5.** Summary of results from the Computer System Usability questionnaire (CSQU). Results are presented in four categories; (OVERALL: all 18 questions), system usefulness (SYSUSE: question 1–8), information quality (INFOQUAL: questions 9–15) and interface quality (INTERQUAL: questions 16–18). Questions were answered on a 7 point Likert scale.

**CSUQ**	**Mean**	**Median**	**Standard Deviation**
**OVERALL**	5.00	4.76	1.00
**SYSUSE**	5.34	5.56	1.03
**INFOQUAL**	4.73	4.43	0.94
**INTERQUAL**	4.63	4.33	1.50
